# Study on the Changes in Volatile Flavor Compounds in Whole Highland Barley Flour during Accelerated Storage after Different Processing Methods

**DOI:** 10.3390/foods12112137

**Published:** 2023-05-25

**Authors:** Wengang Zhang, Xijuan Yang, Jie Zhang, Yongli Lan, Bin Dang

**Affiliations:** 1 Academy of Agriculture and Forestry Sciences, Qinghai University, Xining 810016, China; 2017990098@qhu.edu.cn (W.Z.); 2007990025@qhu.edu.cn (X.Y.); 2015990070@qhu.edu.cn (J.Z.); 2 Key Laboratory of Qinghai Province Tibetan Plateau Agric-Product Processing, Qinghai University, Xining 810016, China; 3 Laboratory for Research and Utilization of Qinghai Tibet Plateau Germplasm Resources, Qinghai University, Xining 810016, China; 4 College of Food Science and Engineering, Northwest A & F University, Yangling 712100, China; yonglilan@nwsuaf.edu.cn

**Keywords:** highland barley, heat processing, volatile compounds, flavor deterioration, gas chromatography-mass spectrometry (GC-MS)

## Abstract

The effect of heat processing on the flavor characteristics of highland barley flour (HBF) in storage was revealed by analyzing differences in volatile compounds associated with flavor deterioration in HBF using GC-MS identification and relative odor activity values (ROAVs). Hydrocarbons were the most abundant in untreated and extrusion puffed HBFs, while heterocycles were found to be the most abundant in explosion puffed, baked, and fried HBFs. The major contributors to the deterioration of flavor in different HBFs were hexanal, hexanoic acid, 2-pentylfuran, 1-pentanol, pentanal, 1-octen-3-ol, octanal, 2-butyl-2-octanal, and (E,E)-2,4-decadienal. Amino acid and fatty acid metabolism was ascribed to the main formation pathways of these compounds. Baking slowed down the flavor deterioration in HBF, while extrusion puffing accelerated the flavor deterioration in HBF. The screened key compounds could predict the quality of HBF. This study provides a theoretical basis for the regulation of the flavor quality of barley and its products.

## 1. Introduction

Highland barley (*Hordeum vulgare* L. *var*. *nudum Hook*. f.) is a gramineous barley crop of the genus barley. It is also known as highland barley due to the separation of its lemma from its caryopsis. Highland barley is rich in starch, protein, dietary fiber, β-glucan, vitamins, minerals, and other nutrients, and it exhibits nutritional qualities of “three highs and two lows” [1,2]. In recent years, there has been increasing interest in the health benefits of highland barley, and its use in the food industry is growing [3]. Long-term intake of highland barley has been shown to positively affect the prevention and alleviation of certain diseases, such as obesity, type II diabetes, hypertension, atherosclerosis, cardiovascular disease, and colon cancer [4,5]. In the food production industry, highland barley is mainly added to various food products in the form of barley flour, and the amount can reach 30% to 60% [5]. However, poor storage stability, a short storage life, and a tendency towards rancidity have limited the development of the highland barley industry. Thus, approaches to extend the shelf life of highland barley through processing are key issues that need to be addressed.

Different heat processing treatments can effectively improve the texture, aroma, and storage stability of grain flours [6]. Changes in freshness during the storage of highland barley flour are most evident as changes in flavor, which often determine the overall sensory characteristics of the analyzed product [7]. The volatile compounds in fresh highland barley flour, mainly from lipid oxidation and the degradation of proteins, carbohydrates, and amino acids, include alcohols, esters, aldehydes, ketones, and alkanes [8]. These compounds vary in aroma type and activity and synergistically constitute the unique flavor of highland barley flour [9]. During heat processing, highland barley is further subjected to various reactions such as lipid oxidation, Maillard, and caramelization, which usually results in a greater abundance of heterocycles and aldehydes and a significant reduction in acids, alcohols, and ketones [10]. Concurrently, heat processing also partially passivates antinutritional factors and lipases, reduces the formation of undesirable flavor compounds due to the oxidation of unsaturated fatty acids, improves pregelatinization, and gives the flour a pleasant roasted aroma, which are important for improving the processing quality and extending the shelf life of highland barley flour [11,12]. Wang et al. found that hot steam treatment could inactivate lipase, lipoxygenase, and peroxidase in buckwheat, thus reducing the production of lipid oxidation markers such as 3-methylbutane and hexanal and thereby delaying the flavor deterioration of stored buckwheat noodles [13]. Extrusion puffing treatment was found to slow down oxidative flavor production during the storage of millet compared to cooking [14]. Jiao et al. showed that roasting treatments based on hot air-assisted RF heating technology could improve the quality and extend the shelf life of nut products such as peanuts [15]. These studies provide new ideas for improving the flavor quality and shelf life of highland barley flour.

At present, the identification of volatile flavor compounds in highland barley and their changes during processing have not been studied much. Tatsu et al. identified 22 and 23 odor-active compounds in highland barley and hulled barley tea, respectively, which mainly included 2-methoxyphenol, trans-isobutanol, 2-acetyl pyrazine, 2-acetyl-1-pyrroline, and 3-methylbutyraldehyde [10]. The main undesirable flavor compounds in cooking barley were reported to be hexanal, (E)-nonenal, (E,E)-2,4-nonadienal, and (E,E)-2,4-decadienal [16]. The sensory quality of barley could be improved by using hot steam treatment to reduce aldehydes (hexanal and (E,E)-2,4-dodecenal) and acids (acetic and hexanoic acids) [9]. However, there is still uncertainty regarding the effects of different heat processing methods on the characteristic flavor and the mechanisms of flavor deterioration in highland barley flour during storage. This study used GC-MS detection and relative odor activity values (ROAVs) to investigate the effects of different processing methods (extrusion puffing, explosion puffing, baking, and frying) on the volatile flavor compounds in highland barley flour. By identifying the key volatile compounds involved in flavor deterioration and the processing methods that can help extend the shelf life of highland barley flour, the study provides a theoretical basis for regulating the flavor quality of highland barley products.

## 2. Materials and Methods

### 2.1. Chemicals and Materials

Kunlun 15 white highland barley was provided by the Qinghai Academy of Agriculture and Forestry Sciences. The test material was planted in 2021 in the experimental field of Qinghai Academy of Agriculture and Forestry Sciences (Xining, Qinghai) (36°67′ N 101°77′ E, 2300 m above sea level). 2-octanol (≥99.5% purity) was purchased from TCI. The test water was deionized.

### 2.2. Preparation of Highland Barley Flour by Different Processing Methods

Highland barley seeds were cleaned, de-mixed, and then processed in different ways. The processing parameters for different treatments in this study were determined through pre-experimental optimization of the preparation conditions for mature highland barley flour. A sample of untreated highland barley flour (Y) was obtained by crushing highland barley seeds using a XL-10B buckling and swinging small crusher (Tianjin Xinhua Instrument Factory, Tianjin, China) and passing them through a 60-mesh sieve. The highland barley flour was extruded using a DZ65-II twin-screw extrusion puffing machine (Jinan Saixin Machinery Co., Ltd., Jinan, China) at a moisture content of 34%, a feed frequency of 22 Hz, a screw speed of 800 r/min, a zone I temperature of 55 °C, a zone II temperature of 180 °C, and a zone III temperature of 160 °C. The extruded material was cooled to room temperature and further powdered through a 60-mesh sieve to obtain the extrusion puffed sample (J). To obtain explosion puffed highland barley flour (Q), 1000 g of highland barley seeds with a moisture content of 10% was added to a XSS-QPD explosion puffing machine (Wuhan Xinshishang Food Machinery Co., Ltd., Wuhan, China) with rotary heating to a pressure of 1.25 MPa after about 7 min, and the valve was opened thereafter. The puffed material was collected and powdered through a 60-mesh sieve to obtain the sample. To obtain baking barley flour (H), highland barley seeds were first soaked for 6 h in water to reach a moisture content of 40%, then evenly spread on a baking tray with a thickness of 0.5 cm and baked in a CK-2 far-infrared food baking oven (Guangzhou Maisheng Baking Equipment Co., Ltd., Guangzhou, China) for 20 min at 150 ± 5 °C for the primer and 170 ± 5 °C for the surface. Finally, the baked highland barley was powdered through a 60-mesh sieve to obtain the sample. For fried barley flour (C), highland barley seeds were first soaked for 6 h in water to reach a moisture content of 40%, then added to a CH50 constant temperature automatic stir fryer (Henan Hui’an Machinery Equipment Co., Ltd., Zhengzhou, China) and fried at 105 ± 5 °C for 10 min. After cooling to room temperature, the fried highland barley was powdered through a 60-mesh sieve to obtain the sample.

### 2.3. Accelerated Storage Tests

Lipid oxidation is an important cause of flavor deterioration in whole grain flour. Thus, we chose accelerated storage conditions for this study by referring to a relevant research report on lipid oxidation during the storage of matured whole grain flour [17]. The accelerated storage test conditions were as follows: 500 g of highland barley flour from the different treatments was stored in woven flour bags (polyethylene) at 50 ± 1% relative humidity and 50 ± 1 °C for 0–84 d to simulate accelerated storage conditions. Appropriate samples were removed every 14 d for the differently treated highland barley flours, repackaged, and stored at −80 °C until gas chromatography–mass spectrometry (GC-MS) test was performed. The control samples were fresh highland barley flour that had not undergone accelerated storage and were directly stored at −80 °C after preparation. Based on a preliminary analysis of the type and relative content of volatile flavor compounds in the analyzed samples, samples stored for 0, 14, and 84 d were selected as representative of the stages of flavor change for subsequent analysis.

### 2.4. The Gas Chromatography–Mass Spectrometry Detection of Volatile Flavor Compounds of Highland Barley Flour

Different samples of highland barley flour (300 mg) were placed in 20 mL headspace bottles. Ten microliters of 2-octanol was added as an internal standard. The volatile flavor compounds were analyzed using a 7890B gas chromatograph, 5977B mass spectrometer, and DB-Wax chromatographic column (30 m × 250 μm × 0.25 μm; Agilent Technologies Inc., Santa Clara, CA, USA). The GC conditions were: extraction temperature, 60 °C; preheating time, 15 min; extraction time, 30 min; resolution time, 4 min; splitless mode; and carrier gas, helium. The column flow rate was 1 mL/min, the sample inlet temperature was 250 °C, and the quadrupole temperature was 150 °C. The temperature rise program of the column box was 40 °C for 4 min, followed by 5 °C/min at 245 °C for 5 min. The mass spectrometry conditions included: ionization voltage, −70 eV; ion source temperature, 230 °C; transmission line temperature, 250 °C; mass scan range, *m*/*z* 20–400; and solvent delay, 0 min.

### 2.5. Analysis of Key Volatile Flavor Compounds

The ROAV method was used to evaluate the contribution of different volatile compounds to the overall flavor of the samples [18]. The greater the ROAV, the greater the contribution of the compound to the overall flavor of the sample. The components that contributed most to the overall flavor of the sample were defined as having ROAVs = 100; herein, all components in the sample had ROAVs ≤ 100. The components with ROAV ≥ 1 were the key flavor compounds in the sample. The components with 0.1 ≤ ROAV < 1 had a modifying effect on the overall flavor of the sample.

The ROAV was calculated according to Equation (1):

(1) $$\mathrm{ROAVi}\approx 100\times \frac{\mathrm{Ci}}{\mathrm{Cs}}\times \frac{\mathrm{Ts}}{\mathrm{Ti}}$$

where Ci is the relative content of volatile compound i (%); Ti is the sensory threshold of volatile compound i (μg/kg); Cs is the relative content of the component contributing most to the overall flavor of the sample (%); and Ts is the sensory threshold of the component contributing most to the overall flavor of the sample (μg/kg).

### 2.6. Data Processing

The MS data were subjected to peak extraction, baseline correction, deconvolution, peak alignment, and MS matching using Chroma TOF software (V4.3x, LECO) and the National Institute of Standards and Technology (NIST) library for quantitative analysis based on relative peak areas. Data were statistically analyzed using Microsoft Excel 2007, Origin 2019b was used for component analysis and graphing, and Simca 14.1 was used for orthogonal partial least squares-discriminant analysis (OPLS-DA). Values were expressed as the mean ± standard deviation (SD) resulting from replicate samples. The annotation and metabolic pathways of differential metabolic volatile compounds in highland barley flour were performed used the KEGG database (http://www.kegg.jp (accessed on 23 September 2021)).

## 3. Results and Discussion

### 3.1. Volatiles in Different Highland Barley Flour

**Figure 1:**
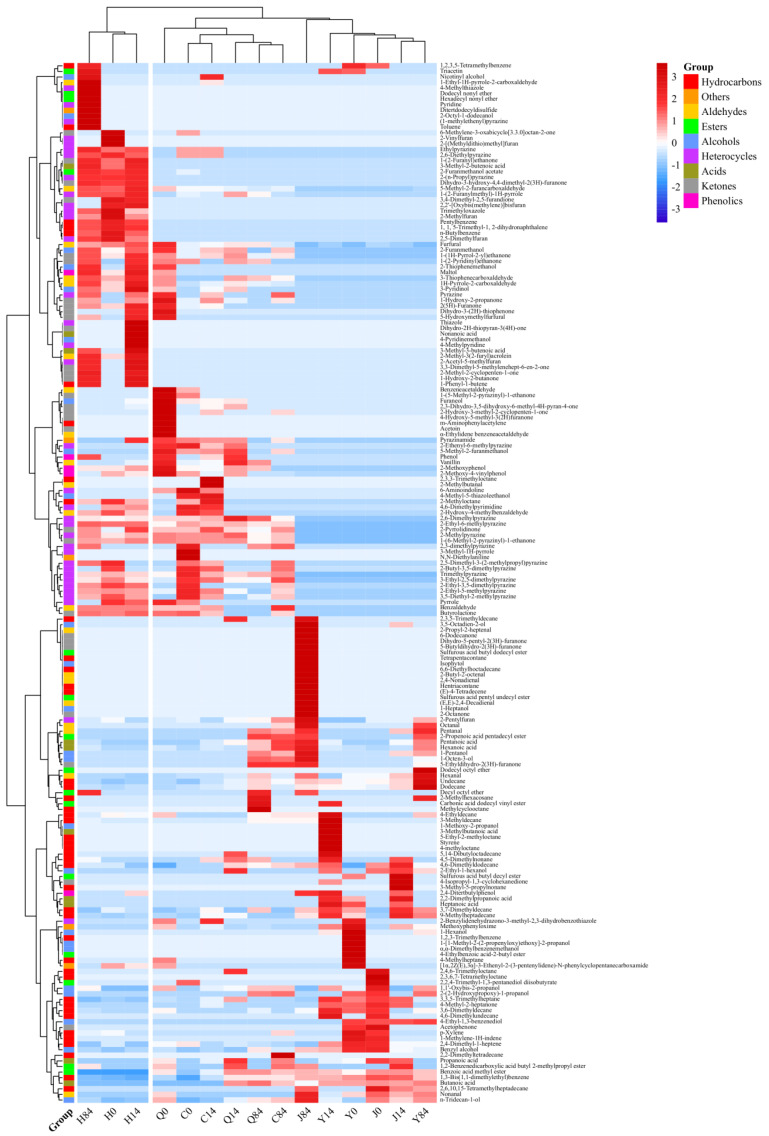
Heatmap of volatile flavor compounds in stored highland barley flour after different heat processing methods. Y0, Y14, Y84: unprocessed samples stored for 0, 14, and 84 d, respectively. Q0, Q14, Q84: explosion puffed samples stored for 0, 14, and 84 d, respectively. J0, J14, J84: extrusion puffed samples stored for 0, 14, and 84 d, respectively. H0, H14, H84: baked samples stored for 0, 14, and 84 d, respectively. C0, C14, C84: fried samples stored for 0, 14, and 84 d, respectively.

A total of 186 volatile metabolites, including alcohols, esters, aldehydes, ketones, acids, heterocycles, phenols, and hydrocarbons, were identified in different samples by GC-MS analysis. The distribution of these compounds is shown in Figure 1, according to which the untreated group was most enriched with hydrocarbons, which were more abundant after extrusion puffing. The heterocyclic compounds were most abundant in the explosion puffed, baked, and fried groups. A significant increase in ketones was observed in the explosion puffed group. Zheng et al. studied the effects of different thermal treatment methods on lipid oxidation and flavor compounds in highland barley and found that aldehydes were the most abundant in untreated, infrared-treated, and steamed highland barley, while esters were the most abundant in roasted, microwaved, and fried highland barley [19]. These results differed from the results of this study, indicating that the variety and treatment methods as well as the conditions affect the characteristic volatile compounds of highland barley flour. During heat processing, the hydrocarbons formed mainly through the oxidative decomposition of unsaturated fatty acids, free radical reactions (cyclization and conjugation), and heat decarboxylation of saturated fatty acids have a high flavor threshold and are generally not considered to be an important factor affecting food flavor [10,15,20]. Ketones are formed mainly through autoxidation, β-oxidation, and decarboxylation of fatty acids and generally have a pleasant aroma. However, their flavor activity is relatively low, and their contribution to the overall flavor of highland barley flours is likely to be small [6]. Heterocyclic compounds, which are mainly produced by meladation, caramelization, and Strecker degradation reactions between reduced sugars and amino acids, have nutty, roasted, and cocoa aromas and are considered to be “good” flavoring substances for the samples [15]. With the increase in storage time, the number of highly expressed volatile compounds tended to decrease in the untreated, explosion puffed, and fried groups, whereas their numbers increased in the extrusion puffed and baked groups. At the end of storage, new low-threshold and highly expressed compounds appeared in different groups. These results suggested significant differences in the volatile flavor compounds of highland barley flour after different treatments or at different storage stages and increased flavor deterioration in different highland barley flours at longer storage times.

### 3.2. Differential Volatile Compounds in Different Highland Barley Flour

**Figure 2:**
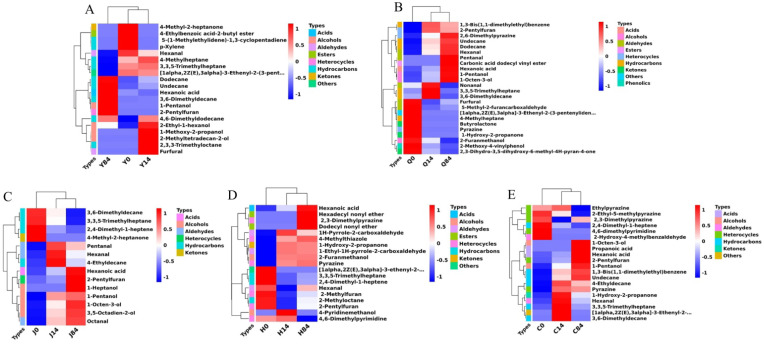
Heatmap of differential metabolic flavor compounds in stored highland barley flour after different processing methods. (**A**–**E**) represent the differential metabolic compounds in unprocessed samples (Y), explosion puffed samples (Q), extrusion puffed samples (J), baked samples (H), and fried samples (C) stored for different times (0, 14, and 84 d), respectively.

Figure 2 showed that 22, 24, 14, 19, and 20 differential metabolic volatile compounds were detected in untreated, explosion puffed, extrusion puffed, baked and fried highland barley flours, respectively, during storage. As storage time increased, the composition of the volatile flavor compounds changed significantly in the groups. In the untreated group, hydrocarbons and alcohols were highly expressed within 14 d of storage, while the low-threshold values of hexanoic acid, 1-pentanol, and 2-pentylfuran were significantly up-regulated at the end of storage. 1-Pentanol and 2-pentylfuran have been reported to be important indicators reflecting lipid oxidation in grains, and hexanoic acid has been associated with undesirable flavor formation in highland barley [9,21]; these compounds may be an important cause of deteriorated flavor in untreated highland barley flour. In the explosion puffed group, the levels of heat-processed characteristic aroma compounds such as pyrazine, 2-methoxy-4-vinylphenol, and 5-methyl-2-furancarboxaldehyde were reduced during storage, while those of low-threshold compounds such as hexanal, pentanal, hexanoic acid, 1-pentanol, and 1-octen-3-ol were increased [6,21]. The significantly upregulated, low-threshold compounds in the explosion puffed group were closely related to lipid oxidation, where hexanal had a grassy and fatty flavor and 1-octen-3-ol had an ‘odor’; these compounds may be the main source of flavor deterioration in explosion puffed highland barley flour [9,14]. In the extrusion puffed group, the highly expressed compounds were mainly hydrocarbons at the beginning of storage. After 14 d of storage, pentanal, hexanal, and 4-methyldecane were significantly upregulated, whereas after 84 d of storage, hexanoic acid, 2-pentylfuran, 1-heptanol, 1-pentanol, 1-octen-3-ol, 3,5-octadien-2-ol, and octanal were significantly upregulated, suggesting that these compounds may be the primary cause of flavor deterioration in extruded highland barley flour. In the baked group, the relative contents of 2-methylfuran, 2-pentylfuran, and 4,6-dimethylpyridine were significantly decreased during storage, while those of hexanoic acid, hexadecyl nonyl ether, 2,3-dimethylpyrazine, and dodecyl nonyl ether were significantly enhanced, suggesting that the loss of some of the characteristic roasted flavors in baked highland barley flour was accompanied by an increase in hexanoic acid content after long-term storage. This significant increase in hexanoic acid and hexanal contents may have contributed more to the deterioration of flavor in baked highland barley flour [9]. At the beginning of storage, the highly expressed compounds in the fried group were mainly heterocyclic, with significant upregulation of 1-hydroxy-2-propanone and hexanal after 14 d of storage and 1-octen-3-ol, propionic acid, pentanoic acid, 2-pentylfuran, and 1-pentanol after 84 d of storage. The low-threshold compounds upregulated at the end of storage may have contributed more to the deterioration of flavor in fried highland barley flour [21]. The changes in pyrazines in the different heat-processed samples were generally small, suggesting that the “rancid flavor” may be mainly the result of masking the “good” aromas by low-molecular-weight aldehydes, ketones, acids, and alcohols produced by lipid oxidation rather than the polymerization or degradation of pyrazines. This finding was consistent with the findings of Warner et al. on flavor deterioration in peanuts [22].

In summary, different processing methods and storage times had significant effects on the differential flavor compounds in highland barley flour. Hexanal, pentanal, octanal, 1-pentanol, 1-octen-3-ol, 1-heptanol, 3,5-octadien-2-ol, hexanoic acid, propanoic acid, pentanoic acid, and 2-pentylfuran may be closely related to the deterioration of flavor in highland barley flour. Variations were observed in the distribution and expression of these compounds in different samples.

### 3.3. Analysis of Key Volatile Compounds

As shown in Table 1, 34 volatile compounds with ROAV ≥ 0.1 were detected in different highland barley flours, including 5 alcohols, 1 ester, 9 aldehydes, 1 ketone, 3 acids, 12 heterocycles, and 3 phenols. The distribution of these compound species differed from their ROAVs in different highland barley flours. The explosion puffed, baked, and fried groups had the highest number of key volatile compounds at 0 d of storage (all 10) and the lowest number in the untreated group (4). The largest contributor to flavor in untreated highland barley flour was hexanal, followed by hexanoic acid, mainly expressed as a green aroma [9]. In the explosion puffed, baked, and fried groups, 2,6-dimethylpyrazine was the largest contributor to flavor, followed by 3-ethyl-2,5-dimethylpyrazine, 2-methoxyphenol, and 2-pentylfuran, which mainly conferred the samples nutty, roasted, smoky, and mung bean-like aromas [23,24]. In the extrusion puffed group, hexanal still contributed the most to the flavor, followed by 2-ethyl-1-hexanol, while the contribution of hexanoic acid was lower than in the untreated group but higher than in the other groups. The order of the effect of the different processing methods on the roasted highland barley flavor profile was baked > fried > explosion puffing > extrusion puffing.

At 14 d of storage, the explosion puffed and fried groups had the most key volatile compounds (9), while the untreated group had the fewest (4). Types of the key volatile flavor compounds decreased after storage of the heat-processed highland barley flours. The compound that contributed most to flavor in the untreated group changed to 2-ethyl-1-hexanol and the ROAV of hexanoic acid increased to 50.71, both with a flowery aroma and sweaty flavor, respectively [25]. The biggest contributor to flavor in the explosion puffed, extrusion puffed, baked, and fried groups continued to be 2,6-dimethylpyrazine, accompanied by the appearance of new key volatile compounds. Among these volatile compounds, 3-methylbutyric acid (ROAV = 2.18) appeared in the untreated group; phenol and 2-ethyl-1-hexanol appeared in the explosion puffed group; 1-pentanol (ROAV = 6.53) and pentanal appeared in the extrusion puffed group; propionic acid and benzoic acid methyl ester appeared in the baked group; and phenol and 2-methylbutanal appeared in the fried group. These compounds, of which 3-methylbutyric acid has a sweat and perspiration flavor and 1-pentanol has a grassy flavor, were found to contribute somewhat to the poor flavor of highland barley flour [9,24]. Pentanal, 2-methylbutanal, and propionic acid had fermented, malty, and sweaty flavors, respectively, but their ROAVs were all less than 1 and they had a secondary role in the overall flavor [10,26]. The other compounds in the different groups showed an overall reduction in ROAV. Overall, the degree of flavor deterioration in the different highland barley flours increased with storage time, which was most significant in the extrusion puffed group, followed by the untreated group, and to a lesser extent in the explosion puffed, baked, and fried groups.

After 84 d of storage, different groups had significant increases in the key volatile flavor compounds; the fried group had the greatest increase and the untreated group had the least increase, indicating further flavor deterioration in the highland barley flour. The largest contributor to flavor was again hexanal in the untreated group and 1-octen-3-ol in the extrusion puffed group, while 2,6-dimethylpyrazine remained the largest contributor to flavor in the explosion puffed, baked, and fried groups. In the explosion puffed, baked, and fried groups, 2-ethyl-6-methylpyrazine, 2,3,5-trimethylpyrazine, and 3-ethyl-2,5-dimethylpyrazine showed a variable increase in ROAV, but they were accompanied by a decrease in ROAV for some other pyrazine compounds. The upregulation of some pyrazines may have been generated again in the accelerated storage test. The key volatile compounds of hexanal (4.53–100.00), hexanoic acid (16.06–95.22), 2-pentylfuran (7.12–40.12), 1-pentanol (9.61–33.49), and pentanal (0.74–1.92) in different samples contributed more significantly after 14 d of storage. In addition, six new key volatile compounds were observed, including 1-octen-3-ol (10.94–100.00), octanal (22.50–69.94), pyridine (1.54), 4-methylthiazole (0.89), 2-butyl-2-octenal (2.69), and (E,E)-2,4-decadienal (1.27). Apart from pyridine and 4-methylthiazole, the remaining nine of these compounds were the main constituents contributing to the undesirable flavor of the different stored highland barley flours, and the formation of these compounds in large amounts may be the result of lipid rancidity [14].

The above-mentioned nine key volatile compounds were most abundant in the extrusion puffed group (9), and their ROAVs was generally high, followed by the fried (7) and untreated groups (7), the explosion puffed group (6), and the baked group, which had the least number of species (3) and their ROAVs were the lowest. Wilkins and Scholl used GC-MS to analyze the volatile metabolites of molds growing on barley and found that the main compounds were 1-octen-3-ol, styrene, and 3-methyl-1-butanol, with smaller amounts of 2-pentylfuran, 3-methylmethyl ether, 2-(2-furyl)pentanal, and 2-ethyl-5-methylphenol [27]. Later, Wang et al. found that 1-octen-3-ol, (E)-2-octen-1-ol, 3-nonen-1-ol, (E)-2-nonenal, decanal, 2-undecanone, and 2-methylnaphthalene appeared or increased with longer storage time in steamed millet flour, in which the main cause of the bad odor of the samples was attributed to 1-octen-3-ol and (E)-2-nonenal [14]. Kaneko et al. reported that hexanal, (E)-nonenal, (E,E)-2,4-nonadienal, and (E,E)-2,4-decadienal caused undesirable odors in cooked barley [16]. The volatile components of aldehydes increased during the storage of buckwheat, and hexanal and 3-methylbutanal could be used as markers of lipid oxidation and flavor changes [13]. Our study findings were congruent with these above-mentioned reports. In summary, nine compounds, including aldehydes, alcohols, acids, and furans, were the main causes of flavor deterioration in stored highland barley flour after different processing methods, and the degree of flavor deterioration in the different highland barley flours was in the order of extrusion puffed group > fried group > untreated group > explosion puffed group > baked group.

**Table 1:** Analysis results of key volatile compounds in stored highland barley flour after different processing methods.

No.	Compounds	OT (μg/kg^−1^)	Odorant Description	ROAV														
				Y0	Q0	J0	H0	C0	Y14	Q14	J14	H14	C14	Y84	Q84	J84	H84	C84
1	2-Methylfuran	9	Chocolate, vanilla				3.05					0.95					0.97	
2	2-Methylbutanal	20	Musty, cocoa, pungent, sweet										0.55					
3	Pentanal	20	Fermented, bready, woodsy, fruity								0.63			1.55	0.74	1.92		1.45
4	Hexanal	4.5	Grass-like, green, fatty	100.00	4.53	100.00	6.74	8.18		5.41	100.00		8.13	100.00	27.96	68.44	4.53	15.88
5	Pyridine	7.9	Sour, fishy															1.54
6	2-Pentylfuran	6	Green bean, butter, vegetable		5.66	29.06	12.83	7.70		3.66	5.17	4.22	2.43	12.10	5.67	40.12	7.12	27.03
7	1-Pentanol	5	Grassy, fruity								6.53			9.76	9.61	33.49		17.03
8	2-Methylpyrazine	60	Nutty, cocoa, roasted		4.34		3.49	3.75		1.79		3.93	3.49		1.24		4.61	3.98
9	4-Methylthiazole	20	Nutty, green, vegetable														0.89	
10	Acetoin	55	Sweet, milky, buttery		0.43													
11	Octanal	0.7	Fatty, soapy, green											32.87		69.94		22.50
12	2,6-Dimethylpyrazine	1.5	Chocolate, nutty, roasted		100.00		100.00	100.00		100.00		100.00	100.00		100.00		100.00	100.00
13	2,3- Dimethyl pyrazine	400	Nutty, peanut, butter					0.13										0.11
14	2-Ethyl-6-methylpyrazine	40	Earthy, roasted		1.25		3.37	2.00		0.71		2.80	1.42		0.80		3.06	1.94
15	2-Ethyl-5-methylpyrazine	100	Coffee bean, nutty, roasted		0.21		0.88	1.09		0.11		0.66	0.59				0.70	0.57
16	Nonanal	260	Citrus-like, rose, grassy	0.43	0.12	0.81					0.10			0.14		0.31		
17	2,3,5-Trime-thylpyrazine	400	Nutty, baked potato, roasted peanut		0.17		0.36	0.48				0.23	0.22		0.14		0.25	0.39
18	3-Ethyl-2, 5-dimethylpyrazine	8.6	Roasted, nutty, earthy		5.99		31.44	29.87		4.28		14.46	20.59		5.33		15.41	36.30
19	1-Octene-3-ol	1	Mushroom											10.94	33.33	100.00		71.60
20	2-Ethyl-3, 5-dimethylpyrazine	40	Roasted, chocolate, cacoa-like				1.47	1.23				0.97					0.87	0.69
21	2-Ethyl-1-hexanol	0.8	Floral, citrus, sweet			85.96			100.00	12.05	19.03			8.28				
22	Benzaldehyde	300	Almond, burnt sugar, cherry				0.15					0.13					0.13	0.20
23	Propionic acid	57	Pungent, acidic, cheesy	0.69	0.27	2.23			0.70	0.27		0.39	0.14					0.64
24	Benzoic acid methyl ester	73	Floral, fruity	1.37	0.39	1.91		0.17	1.92	0.21		0.37	0.26	0.33	0.30	0.46		0.61
25	Benzeneacetaldehyde	9	Flora, honey-like		1.29			0.32										
26	2-Butyl-2-octenal	20	Lemony, fruity													2.69		
27	3-Methylbutyric acid	12	Sweaty						2.18									
28	(E,E)-2,4-Decadienal	1.81	Fatty, green													1.27		
29	Hexanoic acid	2.52	Sweaty, sour, fatty	29.00	14.15	16.18	6.54	3.03	50.71	3.08	4.88	6.21	9.80	38.87	20.34	91.37	16.06	95.22
30	2-Methoxyphenol	0.84	Gammon-like, smoky		24.99		9.84	5.93		4.07		12.10	3.92		5.12		8.40	
31	Benzyl alcohol	100	Sweet, flowery	0.58		0.57												
32	Phenol	0.65	Rubber, phenolic	9.46						3.18			2.91				8.51	
33	Furaneol	27.4	Caramel-like, sweet, maltol-like		0.96			0.14					0.10					
34	2-Methoxy-4-vinylphenol	21	Smoky, clove-like		2.03		1.00	0.76		0.31		0.59	0.24		0.07		0.18	0.15

Note: OT is the abbreviation of ‘odor threshold’. Blank means that the compound was not detected or the aroma type or flavor threshold of the substance was not found. The types of aroma and flavor thresholds were mainly obtained from http://www.thegoodscentscompany.com (accessed on 18 March 2022) and the literature [6,9,10,13,25,28,29,30].

### 3.4. Changes in Key Volatile Compounds during Storage

**Figure 3:**
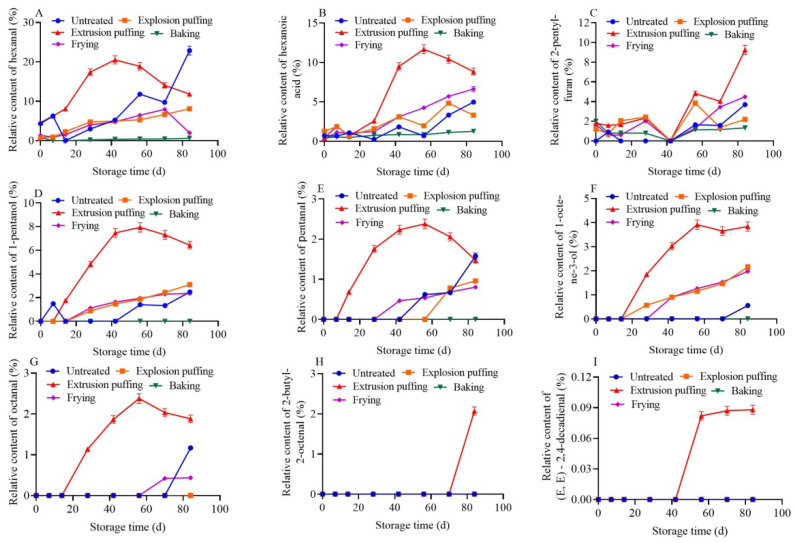
Changes in the relative contents of 9 key volatile compounds during storage of different highland barley flours. (**A**–**I**) represent the relative content changes in hexanal, hexanoic, 2-pentylfuran, 1-pentanol, pentanal, 1-octene-3-ol, octanal, 2-butyl-2-octenal, and (E,E)-2,4-decadienal during storage, respectively.

The relative contents of the nine key volatile compounds were monitored throughout the storage period. The results are shown in Figure 3, according to which the relative content of the nine compounds in the different highland barley flours showed an overall increasing trend with the increase in storage time. The relative content of hexanal in the untreated group increased rapidly and was significantly higher than in the other groups at the end of storage. The relative contents of hexanoic acid, 2-pentylfuran, 1-pentanol, and pentanal increased after 42 d of storage, while the relative contents of 1-octen-3-ol and octanal increased significantly after 70 d of storage. Thus, the turning point for flavor deterioration in the untreated highland barley flour was around 42 d. The relative contents of hexanal, 1-pentanol, and 1-octen-3-ol increased with storage time in the explosion puffed group, while fluctuations were noted in the relative contents of hexanoic acid and 2-pentylfuran. The relative content of pentanal increased rapidly after 56 d of storage, i.e., the turning point for flavor deterioration in the explosion puffed highland barley flour was around 56 d. The relative contents of the nine key volatile compounds were significantly higher in the extrusion puffed group than in the other groups. The relative contents of 2-pentylfuran and 2-butyl-2-octenal increased significantly after 42 and 70 d of storage, respectively, while the relative contents of the other compounds increased significantly after 14 d of storage. These results indicated that extrusion promoted flavor deterioration in highland barley flour, and the turning point was around 14 d. In the baked group, the relative contents of hexanal, hexanoic acid, and 2-pentylfuran increased slowly with storage time, indicating that the flavor deterioration in baked highland barley flour did not have a significant turning point. In the fried group, the relative contents of hexanoic acid and 1-pentanol increased with storage time, while that of hexanal increased and then decreased rapidly. The relative content of 2-pentylfuran began increasing after 42 d of storage, while the relative contents of pentanal and 1-octen-3-ol increased significantly after 28 d of storage, and that of octanal increased slightly at the end of storage. Thus, the turning point for flavor deterioration in fried highland barley flour was around 28 d. It could be seen that the upregulation times for some key volatile compounds differed, which might be due to the different precursors that form these compounds in the samples undergoing different changes during storage. Overall, extrusion accelerated flavor deterioration in highland barley flour, while baking effectively delayed flavor deterioration. We previously conducted research on the storage characteristics of highland barley flour after different processing methods. The results showed that the malondialdehyde levels remained consistently high during accelerated storage of the extrusion puffed and fried groups. The peroxide levels of the extrusion puffed and fried groups varied greatly during accelerated storage, while those of the explosion puffed and baked groups were relatively stable. The fatty acid levels of different treatment groups fluctuated within 42 d of storage and sharply increased after 84 d of storage. The explosion puffed group showed the longest storage time, followed by the baked and fried groups, while the extrusion puffed group had the shortest storage time, which could well support the flavor characterization results in this work [31]. The findings of this study differed from earlier results wherein extrusion processing was beneficial in improving the storage stability of millet and oats, probably due to differences in grain types and their chemical composition [14,23].

In addition, there was a positive correlation between the relative hexanal content and storage time in the untreated (r = 0.811, *p* < 0.05) and explosion puffed (r = 0.971, *p* < 0.05) groups, indicating that the changes in the relative hexanal content were more effective in reflecting the changes in the quality of the untreated and explosion puffed highland barley flours. A linear relationship between the relative content of hexanoic acid and storage time was observed in the fried (r = 0.982, *p* < 0.01) and baked (r = 0.976, *p* < 0.01) groups, indicating that the changes in the relative content of hexanoic acid could better reflect the changes in the quality of fried and roasted baked highland barley flours. In the extrusion puffed group, the linear relationships between the relative contents of 1-pentanol (r = 0.961, *p* < 0.05), pentanal (r = 0.936, *p* < 0.05), 1-octen-3-ol (r = 0.986, *p* < 0.05), and octanal (r = 0.984, *p* < 0.05) and the storage time were good in the range of 14–56 d, indicating that the changes in the relative contents of these four compounds could reflect the changes in the quality of the extrusion puffed highland barley flour over time. In summary, increases in the contents of the nine key volatile compounds during storage indicated increases in the degree of lipid oxidation and deterioration of the different highland barley flours, while the rate of flavor deterioration after different processing methods was in the order of the extrusion puffed group > fried group > untreated group > explosion puffed group > baked group. Some of the key compounds were found to be effective in reflecting the flavor deterioration in highland barley flour after different processing methods.

**Figure 4:**
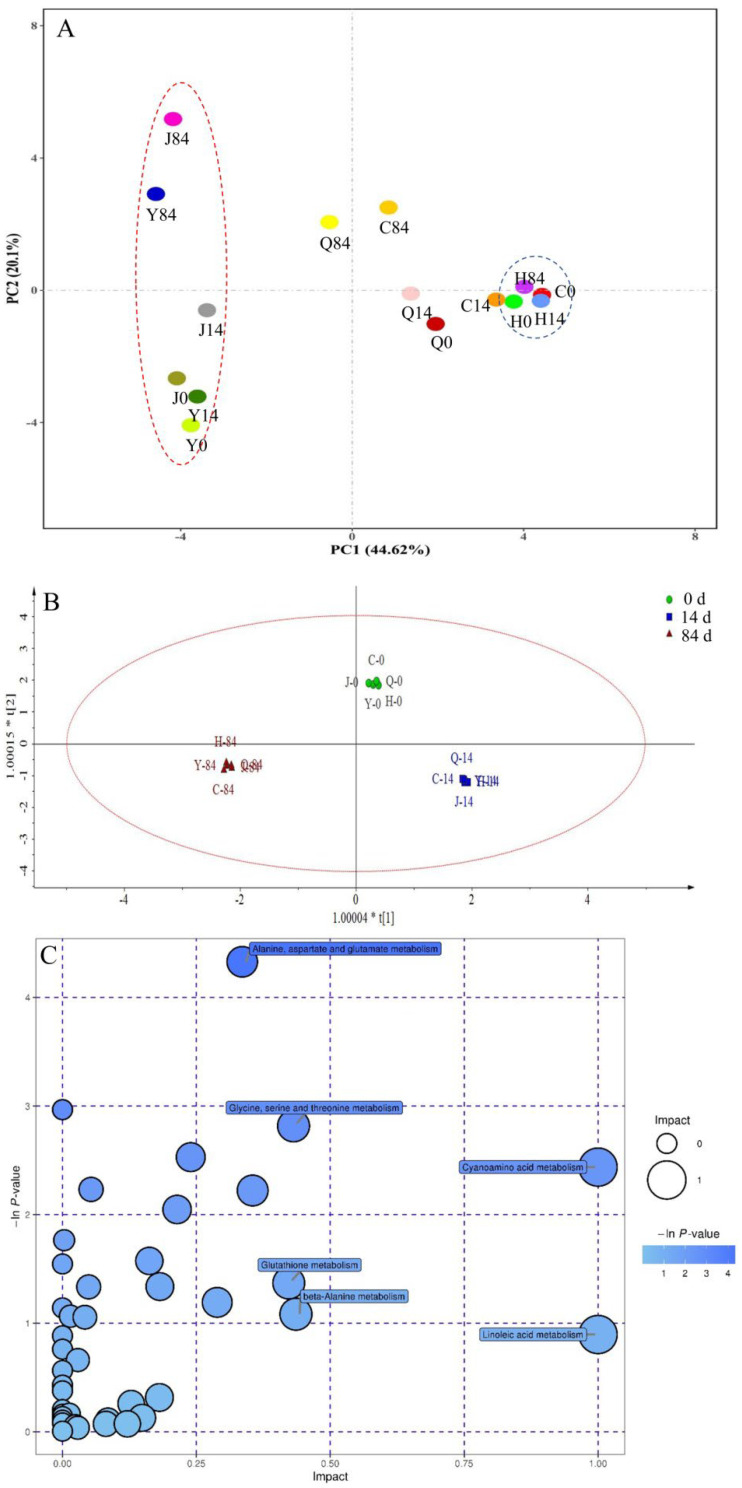
Principal component analysis (PCA) of overall volatile flavor compounds in different highland barley flours (**A**); OPLS-DA scores of key flavor compounds (**B**); Pathway enrichment results of differential metabolites (**C**).

As can be seen from Figure 4A, H0, H14, and H84 were the most concentrated in the principal components analysis (PCA) plot, indicating a relatively higher flavor similarity between samples within the baked group during accelerated storage. The overall flavor change in the other groups started to increase after 14 d of storage and was most obvious in the extrusion puffed group, followed by the untreated group. This finding indicated a faster flavor change in the extrusion puffed group and a slower change in the baked group, which was consistent with the above analysis (Table 1 and Figure 3). However, PCA analysis using the overall volatile compounds provided poor separation of the samples. The key volatile compounds analyzed using the OPLS-DA model showed that the model reached significance (R2X = 0.952, R2Y = 0.993, Q2 = 0.890). The separation of highland barley flours at different storage times (Figure 4B) was significantly higher than that achieved with PCA analysis (Figure 4A), indicating that the key flavor compounds screened in this study could achieve the differentiation and prediction of highland barley flour samples at different stages of storage, and thus provide a theoretical basis for quality control and monitoring of highland barley products.

### 3.5. Analysis of the Formation Mechanism of Key Volatile Compounds

**Figure 5:**
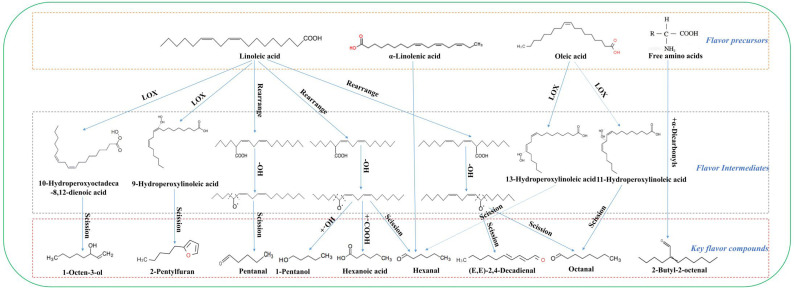
Formation mechanism of 9 key volatile compounds of stored highland barley flour after different processing methods. Note: Structures and metabolic processes of 9 kinds of the key compounds involved in the figure are mainly referred to reports [38,39,41,42].

As shown in Figure 4C, five amino acid metabolism pathways, including the metabolism of alanine, aspartate and glutamate; glycine, serine and threonine; cyanoamino acid; glutathione; and β-alanine, and one fatty acid metabolism pathway of linoleic acid had a significant effect on the formation of volatile flavor compounds at *p* < 0.1 and Impact > 0.4. These results indicated that amino acids and fatty acids are important for the formation of volatile compounds in different highland barley flours and their changes during storage. Highland barley is rich in amino acids and unsaturated fatty acids, and it may provide an important material basis for the metabolism of various volatile compounds in different samples [3]. The metabolic pathways of the nine key compounds identified in this study to be associated with flavor deterioration are shown in Figure 5. The conversion of amino acids to volatile compounds has been confirmed in a variety of plants and plant tissues [32]. In general, amino acids and their derivatives are subject to the removal of amino groups in the reaction catalyzed by branch chain aminotransferases, and the intermediates formed are further converted to aldehydes, alcohols, and esters by decarboxylases and esterification [33]. For example, benzyl alcohol, benzaldehyde, and phenylacetaldehyde may be derived from phenylalanine amino acids, and the chorismate synthase protein is a potential key protein for phenylalanine degradation [34]. Moreover, free amino acids can react with α-dicarbonyl to form Strecker aldehydes, such as benzaldehyde, phenylacetaldehyde, 2-methylbutyraldehyde, and 2-butyl-2-octenal [35].

Unsaturated fatty acids are less stable due to the presence of one or more double bonds and are susceptible to oxidation. Oxidation of oleic, linoleic, α-linolenic, and arachidonic acids with or without enzymatic catalysis (such as lipoxygenase) can form a variety of flavor compounds, including short-chain aldehydes and alcohols, a phenomenon that has been confirmed in grains, fruits, vegetables, vegetable oils, and others [36,37]. Fatty acid oxidation forms small low-threshold aldehydes with grassy, fatty, oily, and fruity flavors, which are common markers of lipid oxidative rancidity and food deterioration [25]. During storage, the lipids in highland barley flour are hydrolyzed by lipase to release unsaturated fatty acids, resulting in high levels of free fatty acids in highland barley flour during the middle and late stages of storage. These free fatty acids are subject to rearrangement, autoxidation, and enzymatic oxidation, usually to form the corresponding hydroperoxides (13-, 11-, or 10-ROOH), followed by the action of hydroperoxide lyase to produce aldehydes, which can be reduced to alcohols by alcohol dehydrogenase [38,39]. The metabolism of unsaturated fatty acids can ultimately produce undesirable flavor compounds, such as 1-octen-3-ol, 1-pentanol, hexanol, heptanol, nonanol, hexanal, nonanal, heptanal, octanal, and (E,E)-2,4-nonadienal, which was further confirmed by the results of this study [40].

## 4. Conclusions

The composition of volatile flavor compounds during storage differed significantly in highland barley flour treated with different processing methods. After heat processing, extrusion puffed highland barley flour formed more hydrocarbons and explosion puffed highland barley flour formed more ketones and heterocycles, while baked and fried highland barley flours formed a large number of heterocycles. With the extension in storage time, there was a dynamic change in the differential metabolic compounds in highland barley flour, flavor deterioration increased, and low-threshold compounds appeared at the end of storage. Extrusion and frying accelerated the flavor deterioration in highland barley flour and the effect was in the order of extrusion puffing > frying, while explosion puffing and baking slowed the flavor deterioration in highland barley flour, in the order of baking > explosion puffing. Five aldehydes (hexanal, pentanal, octanal, 2-butyl-2-octenal, and (E,E)-2,4-decadienal), two alcohols (1-pentanol and 1-octen-3-ol), one acid (hexanoic acid), and one heterocycle (2-pentylfuran) were the key volatile compounds observed to be involved in the flavor deterioration of differently treated highland barley flours. The formation of these compounds mainly involved amino acid metabolism and linoleic acid metabolism. The relative contents of these compounds could be used to reflect the flavor deterioration process in highland barley flour at different processing stages and to distinguish the quality of highland barley flour at different storage times. This study can provide a theoretical basis for understanding the storage quality and implementing flavor quality control of hot-processed highland barley products.

## Data Availability

Data is within the article.
